# Radiotherapy for Cervical Cancer: Technical Advances, Clinical Applications, and Future Perspectives

**DOI:** 10.1111/jog.70555

**Published:** 2026-11-06

**Authors:** Hitoshi Maemoto, Takafumi Toita, Akihiro Nishie

**Affiliations:** ^1^ Department of Radiology, Graduate School of Medicine University of the Ryukyus Okinawa Japan; ^2^ Radiation Therapy Center, Okinawa Chubu Hospital Okinawa Japan

**Keywords:** cervical cancer, image‐guided brachytherapy, intensity‐modulated radiotherapy, intracavitary/interstitial brachytherapy, personalized medicine, radiotherapy

## Abstract

Radiotherapy is an essential treatment modality for cervical cancer and has evolved substantially with advances in image guidance and treatment delivery. This review summarizes recent technological developments in external beam radiotherapy and brachytherapy, their clinical applications, and future directions, with particular consideration of Japanese practice in an international context. In external beam radiotherapy, conventional techniques incorporating central shielding are increasingly being replaced by intensity‐modulated radiotherapy (IMRT) and volumetric‐modulated arc therapy (VMAT). Daily three‐dimensional image‐guided radiotherapy (3D‐IGRT) is essential for the safe implementation of these techniques because of substantial uterine motion and deformation. In brachytherapy, the transition from two‐dimensional point‐based prescription to three‐dimensional image‐guided treatment has enabled individualized assessment of target coverage and doses to organs at risk. Although magnetic resonance imaging‐based brachytherapy is internationally recommended, computed tomography‐based planning predominates in Japan and has achieved favorable outcomes, despite some studies reporting lower cumulative target doses than those recommended internationally. Intracavitary/interstitial brachytherapy (IC/IS BT) further improves dose distribution by combining intracavitary applicators with interstitial needles, particularly when adequate target coverage cannot be achieved with standard intracavitary brachytherapy alone. Future advances may include artificial intelligence (AI)‐assisted treatment planning and adaptive workflows, tissue spacers to overcome anatomical constraints, and the incorporation of genomic, multi‐omics, and radiomics information. Integrating these approaches may enable cervical cancer radiotherapy to progress beyond increasingly safe and precise treatment toward personalized care tailored to individual patient and tumor characteristics.

## 1 Introduction

Cervical cancer remains the fourth most common malignant tumor and the fourth leading cause of cancer‐related death among women worldwide, with approximately 660 000 new cases and 350 000 deaths annually [1]. Persistent human papillomavirus (HPV) infection is its main cause, and cervical cancer is largely preventable through vaccination and screening [2, 3, 4, 5, 6], and its burden remains concentrated in low‐ and middle‐income countries [7]. Japan is an exception among high‐income countries, with a substantial disease burden partly attributable to historically low HPV vaccination coverage [8, 9, 10].

Treatment strategies for cervical cancer continue to advance across disease stages, from less invasive surgery for early‐stage disease to increasingly effective systemic and local treatments for stage IVB or recurrent disease. In stage IVB cervical cancer, retrospective propensity score‐matched analyses, including a recent Japanese multicenter JROSG22‐1/JCOG1088S study, suggest that definitive pelvic radiotherapy, particularly when combined with brachytherapy, may prolong survival in appropriately selected patients, although prospective validation is required [11, 12]. For oligometastatic cervical cancer, stereotactic body radiotherapy (SBRT) has been associated with excellent local control and favorable survival outcomes in selected patients [13].

Radiotherapy has traditionally played an essential role in the management of locally advanced cervical cancer, primarily as a component of concurrent chemoradiotherapy (CCRT) [14]. Treatment strategies in this setting have also continued to evolve, with recent studies demonstrating improved survival through the addition of pembrolizumab [15] and the use of induction chemotherapy before CCRT [16]. In parallel, advances in radiotherapy—including intensity‐modulated radiation therapy (IMRT), three‐dimensional image‐guided brachytherapy (3D‐IGBT), and intracavitary and interstitial brachytherapy (IC/IS BT)—have improved target coverage and organ‐at‐risk (OAR) sparing, enabling increasingly individualized treatment.

This review summarizes technological advances in radiotherapy for cervical cancer, their clinical applications, and future directions, with particular consideration of Japanese practice in an international context.

## 2 Advances in EBRT

### 2.1 Conventional EBRT With Central Shielding (CS)

Definitive radiotherapy for cervical cancer generally consists of EBRT to the whole pelvis followed by high‐dose rate brachytherapy. In patients with FIGO 2018 stage IIIC2 disease, the para‐aortic lymph node region is also included in the treatment target.

Historically, conventional EBRT has been delivered using a four‐field box technique or a combination of anterior–posterior opposed fields. In Japan, CS has traditionally been incorporated during EBRT after the delivery of 30‐40Gy without CS from the era of two‐dimensional (2D) planning [17, 18, 19, 20] to the recent three‐dimensional (3D) planning [21, 22, 23, 24]. The primary purpose of CS was to reduce radiation exposure to the rectum, bladder, and small bowel, which subsequently receives a high radiation dose during brachytherapy.

Since CS uniformly blocks the central pelvic region without accounting for individual variations in tumor extent, uterine position, or the spatial relationship between target and surrounding OARs, accurate assessment of the cumulative dose delivered to the target and OARs has been challenging. Despite this limitation, CS was widely adopted in clinical practice for many years because of its practical feasibility.

Recent advances in radiation delivery techniques and image guidance have enabled a transition from conventional CS‐based EBRT (Figure 1A) to more conformal approaches using IMRT (Figure 1B), which is increasingly becoming the standard EBRT technique for cervical cancer.

**FIGURE 1:**
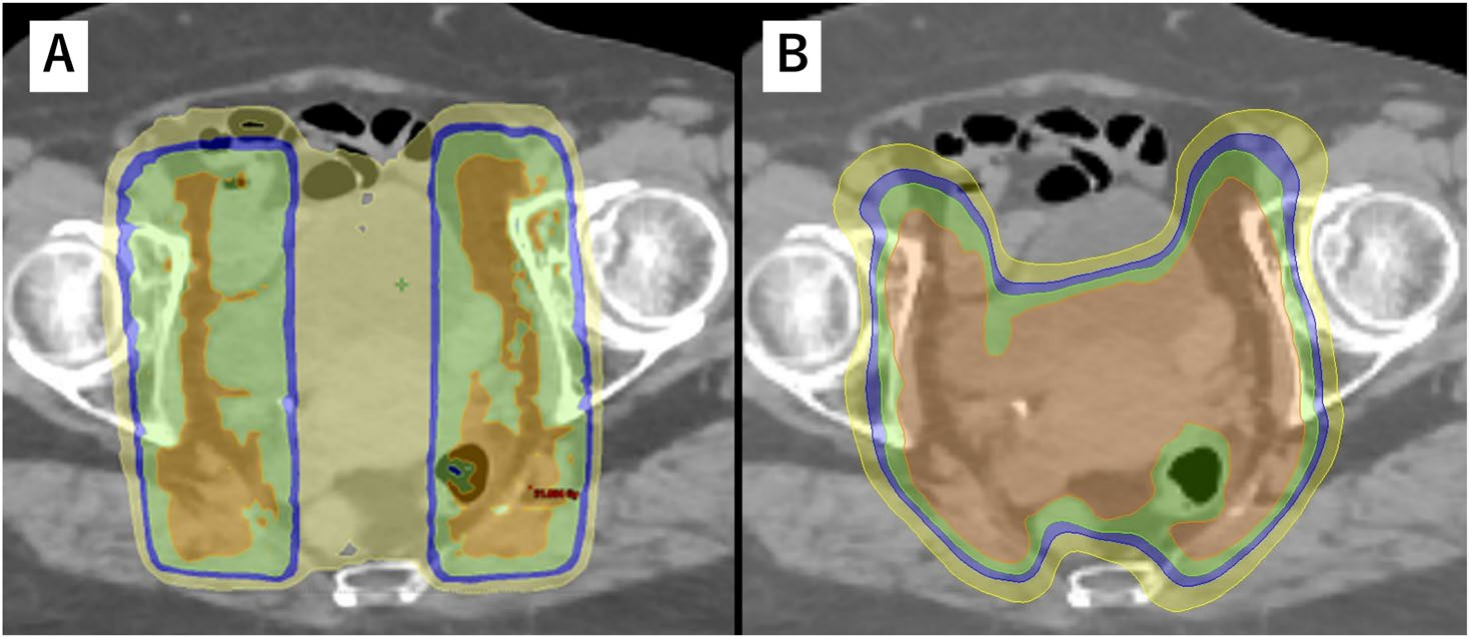
Dose distributions of (A) a composite plan combining four‐field whole‐pelvic irradiation without central shielding and subsequent anteroposterior–posteroanterior (AP–PA) irradiation with central shielding, and (B) intensity‐modulated radiotherapy. Orange, light green, blue, and yellow represent the 100%, 95%, 90%, and 80% isodose levels, respectively.

### 2.2 Challenges in Implementing IMRT

IMRT is a highly conformal radiotherapy technique that was first covered by the Japanese national health insurance system for localized solid tumors in 2008 [25]. Compared with conventional 3D conformal radiotherapy (3D‐CRT), IMRT modulates beam fluence using multileaf collimators (MLC), allowing improved dose conformity to the target while reducing radiation exposure to surrounding OARs (Figure 1).

Early IMRT was typically delivered using five to seven fixed gantry angles. Volumetric‐modulated arc therapy (VMAT) was subsequently developed, enabling continuous modulation of beam intensity during gantry rotation [26]. By achieving comparable or superior dose distributions with substantially shorter treatment times than fixed‐field IMRT [27], VMAT has become widely adopted in clinical practice.

The inherent mobility of the uterus has represented a major obstacle to the implementation of IMRT for cervical cancer because the highly conformal dose distribution of IMRT is sensitive to target positional changes. Previous studies have demonstrated that uterine motion is small in the lateral direction but greater in the craniocaudal and anteroposterior directions, with the uterine corpus exhibiting greater displacement than the cervix [28, 29, 30]. Consistent with these findings, our previous study demonstrated that the mean lateral displacement of the uterine corpus was approximately 3 mm during radiotherapy, whereas the mean displacement reached approximately 8 mm in both the craniocaudal and anteroposterior directions [31].

Such uterine motion can be addressed by expanding the planning target volume (PTV) margin. However, larger margins lead to increased radiation exposure to surrounding OARs at the expense of the dose conformity that represents a principal advantage of IMRT. A more rational strategy is to employ image‐guided radiotherapy (IGRT), which uses image guidance before each treatment fraction to accurately localize the target and correct for positional deviations [32]. This approach enables the dosimetric benefits of IMRT to be maintained without excessively large PTV margins.

### 2.3 Advances in IGRT

To overcome this limitation, IGRT has become indispensable for the safe and accurate delivery of IMRT. IGRT is an image‐guided positioning technique that uses images acquired on the radiotherapy unit to verify the positions of the patient and the target, allowing the planned treatment position to be reproduced as accurately as possible [32].

Position verification in IGRT is performed using either 2D images acquired from at least two directions or 3D imaging. Before the widespread adoption of 3D image guidance, 2D image matching based on bony anatomy was the standard approach. However, because the uterus moves independently of the bony anatomy, 2D image guidance based on bony anatomy is insufficient for accurately assessing uterine position [33]. Furthermore, variations in bladder and rectal filling can displace and deform the uterus and may increase OAR doses [31], underscoring the importance of reproducible organ filling. Consequently, 3D image guidance using megavoltage computed tomography (MVCT) or cone‐beam computed tomography (CBCT) is indispensable for IMRT in cervical cancer [34].

3D‐IGRT using MVCT or CBCT enables organ position to be verified and corrected immediately before each treatment fraction. In addition, the clinical introduction of robotic couches capable of correcting patient positioning in six degrees of freedom based on image guidance has further improved setup accuracy [35]. These technological advances have provided the technical foundation for the safe and accurate implementation of IMRT in cervical cancer.

### 2.4 Clinical Benefits of IMRT

The clinical benefits of IMRT have been most clearly demonstrated in the postoperative setting. A randomized study comparing IMRT with conventional 3D‐CRT has shown significant reductions in acute gastrointestinal and genitourinary toxicity [36]. Pelvic bone marrow‐sparing IMRT has also been associated with reduced hematologic toxicity [37]. Improvements have also been reported in late toxicity, including a reduction in grade ≥ 2 late gastrointestinal toxicity from 42.2% with 3D‐CRT to 21.1% with IMRT [38] and better preservation of urinary function beyond 3 years after treatment [39]. However, these randomized trials were primarily designed to evaluate toxicity and did not establish the non‐inferiority of IMRT in terms of oncologic outcomes. This question was subsequently addressed in JCOG1402, a multicenter single‐arm confirmatory trial of postoperative CCRT using IMRT for patients with high‐risk cervical cancer. The 3‐year relapse‐free survival rate was 79.9% (90% confidence interval, 75.0%–83.9%), establishing the oncologic non‐inferiority of IMRT‐based treatment [40].

Evidence supporting IMRT in the definitive treatment setting is less robust than that in the postoperative setting. A prospective randomized study by Gandhi et al. demonstrated that IMRT significantly reduced acute gastrointestinal toxicity compared with conventional pelvic radiotherapy without compromising early disease control [41]. However, large randomized Phase III trials directly comparing IMRT with conventional radiotherapy for definitive chemoradiotherapy are still lacking.

Nevertheless, based on accumulating evidence demonstrating improved dose conformity and reduced treatment‐related toxicity, contemporary international guidelines recommend IMRT/VMAT with daily IGRT as the preferred external beam technique for the definitive treatment of cervical cancer [42, 43].

In contrast to the widespread international adoption of IMRT for cervical cancer, its advantages over conventional 3D‐CRT may historically have been perceived as less pronounced in Japan. This may partly be explained by the use of CS, which had already been incorporated into conventional pelvic radiotherapy to reduce irradiation of the bladder, rectum, and small bowel. Consequently, the additional reduction in radiation exposure to OARs expected with IMRT may have appeared smaller than that observed in Western studies comparing IMRT with conventional four‐field whole‐pelvic irradiation without CS. In addition, the relatively smaller pelvic capacity of Japanese patients and the close proximity of the small bowel to the uterus [44] may limit the extent of bowel sparing achievable with IMRT.

In this context, direct evidence comparing IMRT with the CS technique traditionally used in Japan remains limited, and the clinical impact of IMRT over the conventional Japanese approach has not yet been fully established. Nevertheless, Japanese studies have demonstrated the feasibility of combining EBRT without CS with 3D‐IGBT [45, 46]. In this setting, IMRT is particularly suitable because it enables individualized dose delivery according to the actual extent of the target while maintaining coverage of the entire target volume and limiting the dose to surrounding OARs without the need for uniform CS. This treatment strategy provides a rationale for the use of IMRT without CS.

Evidence from Japan has recently strengthened. Although JCOG1402 was conducted in the postoperative setting and therefore did not directly address the benefit of IMRT over CS‐based definitive radiotherapy [47], it prospectively demonstrated the oncologic non‐inferiority and safety of IMRT‐based CCRT in Japanese patients [40].

Overall, accumulating clinical evidence and advances in image guidance and treatment planning have supported the increasing adoption of IMRT for cervical cancer in Japan, and its use is likely to expand further as these technologies continue to evolve.

## 3 Advances in Brachytherapy

### 3.1 From 2D to 3D‐IGBT

Just as IGRT enabled the full clinical implementation of IMRT, advances in image guidance have dramatically improved brachytherapy. A small Phase I/II study conducted in Japan suggested that SBRT boost may represent an important treatment option for intracavitary brachytherapy (ICBT)‐ineligible patients [48]. At present, however, brachytherapy is an essential component of definitive radiotherapy for cervical cancer, as its omission is associated with significantly inferior survival outcomes [49, 50], and technological innovations in this field have contributed to improvements in treatment outcomes.

For many decades, two‐dimensional intracavitary brachytherapy (2D‐ICBT) was the standard approach. In 2D‐ICBT, although the applicator and bony structures can be identified on x‐ray images, it is not possible to evaluate the extent of the tumor or the spatial relationship between the uterus and OARs. Consequently, radiation dose was prescribed to Point A, which had been widely adopted since the 1950s [51]. Point A is defined as a point located 2 cm superior to the external cervical os and 2 cm lateral to the uterine tandem in the plane of the uterus. Point A was devised as a point representative of the tumor that offers high reproducibility across patients. However, even before the advent of modern imaging modalities, it was evident that Point A did not consistently represent the true extent of the tumor in individual patients.

Since the 2010s, 3D‐IGBT using CT or MRI has been widely adopted in clinical practice [52]. In 3D‐IGBT, the high‐risk clinical target volume (HR‐CTV) and OARs are individually contoured for each patient, and dose prescription and evaluation are based on patient‐specific target volumes and OARs. This represented a paradigm shift from empirical point A‐based prescription to image‐guided individualized treatment. By enabling dose escalation to the target while reducing radiation doses to OARs, 3D‐IGBT has been associated with improved oncologic outcomes and reduced treatment‐related toxicity [24, 53, 54, 55, 56, 57].

In Europe, where the adoption of 3D‐IGBT began early, MRI‐based contouring of the HR‐CTV using T2‐weighted images has been recommended according to the Groupe Européen de Curiethérapie/European Society for Radiotherapy and Oncology (GEC‐ESTRO) consensus guidelines [58]. In contrast, MRI‐based 3D‐IGBT has been less widely adopted in East and Southeast Asia, including Japan, where CT‐based 3D‐IGBT has become the predominant approach [59].

Recognizing the limited availability of MRI in many regions, the Indian Brachytherapy Society, GEC‐ESTRO, and the American Brachytherapy Society subsequently developed international recommendations for CT‐based target contouring, adapting MRI‐based concepts to different clinical and imaging environments [60]. A recent validation study comparing CT‐based contouring according to these recommendations with MRI‐based contouring reported comparable target dose parameters despite some differences in target dimensions and OAR delineation [61].

In Japan, the Japanese Radiation Oncology Study Group (JROSG) previously proposed CT‐based contouring guidelines for HR‐CTV [62], and several Japanese studies have reported favorable clinical outcomes with CT‐based 3D‐IGBT despite its inferior soft tissue contrast [21, 23, 24, 54].

In MRI‐based 3D‐IGBT, cumulative HR‐CTV doses of approximately 85 Gy or higher have been commonly reported [53, 55, 57]. Consistently, the European Society of Gynecological Oncology (ESGO)/ESTRO/European Society of Pathology (ESP) guidelines recommend a cumulative HR‐CTV D90 of 85–90 Gy in equivalent dose in 2 Gy fractions (EQD2) as the target dose for definitive treatment [63]. In contrast, Japanese studies have reported favorable local control and a low incidence of severe late toxicities with lower cumulative doses of approximately 65–75 Gy [21, 22, 23, 64, 65, 66].

Several factors may account for the lower cumulative doses reported in Japanese studies. First, the prescribed dose itself may have actually been lower in clinical practice. Compared with Western populations, Japanese patients generally have a smaller pelvic cavity, with the small bowel located closer to the uterus [44], making dose escalation more challenging because of concerns regarding severe gastrointestinal toxicity.

On the other hand, it has also been suggested that the reported dosimetric parameters may underestimate the actual dose delivered to the target. CT‐based 3D‐IGBT has a tendency of contouring larger HR‐CTV than MRI‐based planning, resulting in lower DVH parameters, such as D90, despite similar actual delivered doses [67]. Furthermore, the historical use of CS during EBRT in Japan should also be considered. Because the contribution of CS to the cumulative HR‐CTV dose cannot be accurately quantified, the actual delivered dose may have been underestimated in conventional Japanese treatment protocols [68].

More recently, however, Japanese studies have reported clinical outcomes with treatment strategies combining EBRT without CS and 3D‐IGBT [45, 46]. These studies demonstrated that favorable local control could still be achieved with an HR‐CTV D90 of approximately 70 Gy, particularly in patients with T1–T2 disease [46]. Taken together, these findings suggest that the optimal HR‐CTV dose may differ from current international recommendations in carefully selected Japanese patients, underscoring the need for individualized brachytherapy prescription rather than applying a universal dose recommendation across different populations.

### 3.2 IC/IS BT

The widespread adoption of 3D‐IGBT has revealed the dosimetric limitations of standard ICBT using tandem‐and‐ovoid or tandem‐and‐cylinder applicators. In particular, in patients with bulky locally advanced tumors or residual parametrial extension, standard ICBT may not provide adequate coverage of the entire HR‐CTV without exceeding acceptable dose constraints for OARs.

In brachytherapy, the dose decreases steeply with increasing distance from each source position. Therefore, when the target cannot be adequately covered by the dwell positions available within standard intracavitary applicators, additional dwell positions closer to the areas of tumor extension are required. To overcome this limitation, IC/IS BT, which combines intracavitary and interstitial techniques, has been developed and increasingly adopted [69]. In IC/IS BT, one or more interstitial needles are inserted in addition to the intracavitary applicator, providing additional source dwell positions and allowing more flexible dose distributions tailored to the shape and extent of the tumor (Figure 2).

**FIGURE 2:**
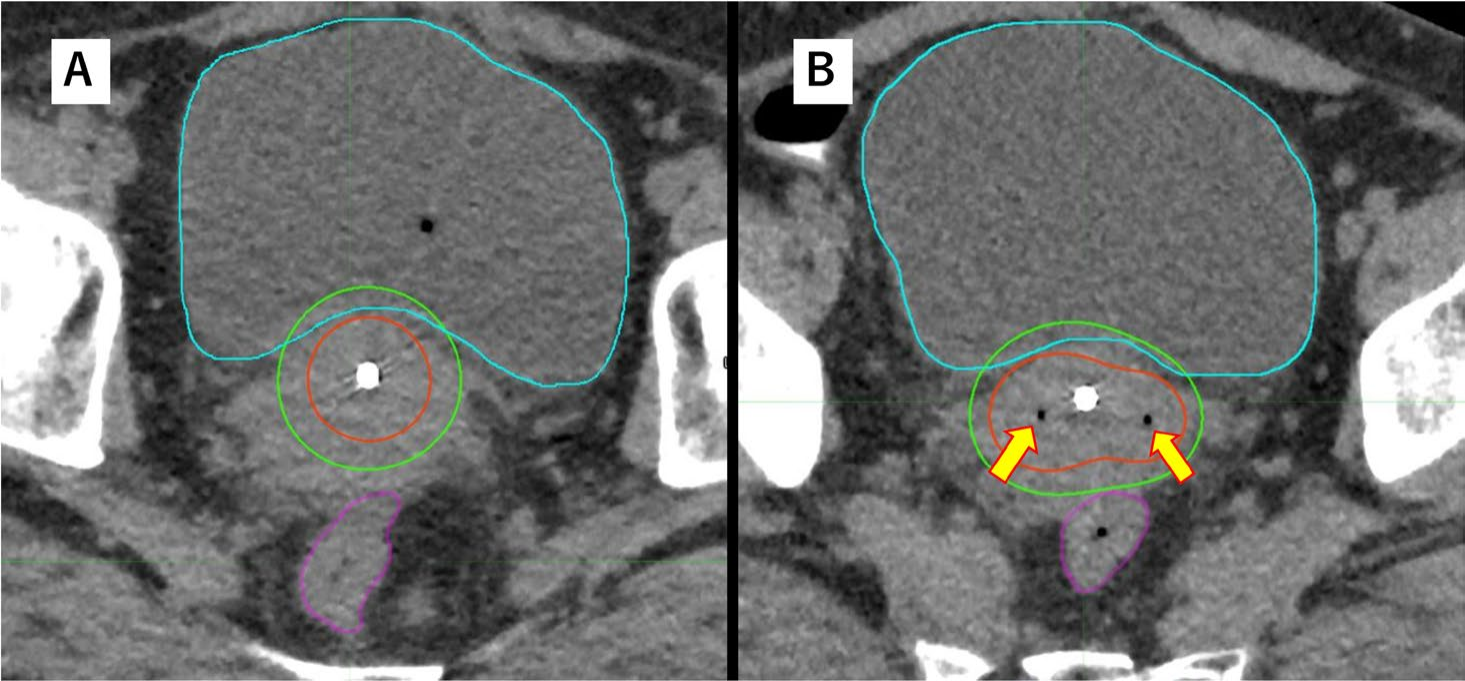
Dose distributions in the same patient undergoing (A) intracavitary brachytherapy (ICBT) alone and (B) intracavitary/interstitial brachytherapy (IC/IS BT). The round white structure within the uterine cavity represents the cross‐section of the tandem applicator, and two yellow arrows in panel B indicate the positions of the interstitial needles. The orange and light‐green lines represent the 150% and 100% isodose lines, respectively. The bladder and rectum are contoured in light blue and purple, respectively. Compared with ICBT alone, IC/IS BT improves target coverage without increasing the doses to the bladder and rectum.

Even the addition of a small number of interstitial needles can substantially improve dose distribution. The RetroEMBRACE study, a landmark large‐scale multicenter study evaluating IGBT for locally advanced cervical cancer, demonstrated that institutions systematically implementing IC/IS BT had a 9‐Gy higher HR‐CTV D90 (92 vs. 83 Gy) without increasing doses to OARs. Furthermore, among patients with large tumors (HR‐CTV ≥ 30 cm^3^), systematic use of IC/IS BT was associated with an approximately 10% improvement in 3‐year local control, highlighting the clinical value of IC/IS BT, particularly in patients for whom adequate target coverage cannot be achieved with standard ICBT [70].

Clinical evidence supporting the utility of IC/IS BT has also accumulated in Japan. A retrospective comparison of ICBT and IC/IS BT, predominantly in Japanese patients, demonstrated comparable 4‐year local control between the two groups (89.3% with ICBT vs. 86.8% with IC/IS BT, *p* = 0.314), despite more adverse clinical factors in patients treated with IC/IS BT, supporting the potential of IC/IS BT to maintain local control in this less favorable patient population [66]. Furthermore, a prospective Phase I/II study conducted in Japan in patients with FIGO Stage IB2–IVA cervical cancer reported a 2‐year progression‐free survival (PFS) rate of 80.7% with IC/IS BT, compared with 64% in a historical ICBT cohort [71].

IC/IS BT involves interstitial needle insertion and may therefore raise concerns regarding procedure‐related complications, including bleeding, infection, and injury to surrounding organs. Dedicated IC/IS applicators incorporate needle‐guiding holes that constrain the insertion position and direction and improve procedural reproducibility compared with freehand needle placement [72]. In an initial clinical series using such an applicator, no needle‐related bleeding or organ penetration occurred during 40 treatment fractions involving 66 needle insertions [73]. In addition, a Japanese study demonstrated that IC/IS BT can be performed safely with transrectal ultrasound guidance [74]. Although direct comparative evidence that these approaches reduce complications remains limited, IC/IS BT has become an important component of modern brachytherapy for cervical cancer, particularly when adequate target coverage cannot be achieved with standard ICBT alone.

The importance of IC/IS BT is also reflected in recent international quality indicators. In the ESGO/ESTRO quality indicators for cervical cancer radiotherapy, the proportion of patients treated with IC/IS BT among those undergoing brachytherapy at each institution has been adopted as an institution‐level indicator, with ≥ 60% defined as the optimal target and ≥ 40% as the minimum required target [75]. These recommendations underscore that IC/IS BT should no longer be regarded as a specialized technique confined to a limited number of highly specialized centers, but rather as an integral component of modern brachytherapy for cervical cancer.

However, the implementation of IC/IS BT increases the complexity of both the procedure and treatment planning, requiring additional resources in terms of personnel, procedural time, image‐guidance capabilities, anesthesia or sedation, and preparedness for procedure‐related complications such as bleeding [76]. Successful implementation therefore requires appropriate training and well‐established clinical workflows. In Japan, efforts to facilitate the wider adoption of IC/IS BT have included hands‐on training programs and educational initiatives [14].

With 3D‐IGBT enabling individualized assessment of target coverage and OAR doses based on tumor anatomy, brachytherapy has evolved beyond dose evaluation toward optimization of source placement itself. IC/IS BT represents a key means of achieving this goal, and establishing the clinical infrastructure to deliver IC/IS BT safely and efficiently to appropriately selected patients is essential for modern cervical cancer radiotherapy.

## 4 Future Directions

### 4.1 Managing Complexity: Artificial Intelligence (AI)‐Assisted Radiotherapy

Diagnostic imaging is one of the areas in which AI has been most extensively integrated into clinical practice, with applications spanning image reconstruction, accelerated image acquisition, and lesion detection [77, 78, 79, 80, 81]. More recently, the role of AI has expanded beyond image analysis to include report generation and broader support for clinical workflows [82].

In radiotherapy, technological advances such as IMRT, 3D‐IGBT, and IC/IS BT have improved treatment precision but have also increased the complexity of contouring and treatment planning, resulting in greater demands on both personnel and time. Furthermore, with the demand for radiotherapy expected to increase, improving workflow efficiency will become increasingly important for the sustainable delivery of high‐quality treatment with limited healthcare resources. In this context, AI‐assisted approaches are increasingly being explored to improve the efficiency of radiotherapy workflows, with autosegmentation already being integrated into clinical practice [83].

Radiotherapy for cervical cancer is particularly well suited to AI‐assisted approaches. Pelvic organs can undergo substantial changes in position and shape depending on their filling status. In addition, the uterus itself may exhibit considerable displacement and deformation during the course of treatment. Changes in body contour due to treatment‐related weight loss or edema may further compromise the validity of the initial treatment plan, necessitating reimaging, recontouring, and replanning. AI‐assisted approaches, including autosegmentation and support for adaptive radiotherapy and replanning, may streamline these time‐consuming processes, reduce variability among planners and institutions, and improve the quality and reproducibility of increasingly complex cervical cancer radiotherapy [84, 85]. In brachytherapy, a recent Japanese planning study using an inverse planning technique showed that uniformly restricting needle dwell times to the conventionally recommended range of 10%–20% could increase OAR doses, highlighting the need for individualized optimization in IC/IS BT [86]. AI‐based approaches are also being explored for target and OAR segmentation and treatment plan optimization [87].

However, the performance of AI models depends on factors such as training data, image acquisition conditions, and patient characteristics, potentially limiting their generalizability across institutions and patient populations. Generalizability and bias remain important challenges in medical AI [88]. Therefore, safe clinical implementation requires not only adequate external validation but also appropriate oversight and final verification by radiation oncologists.

### 4.2 Overcoming Anatomical Constraints: Tissue Spacers

Even with advances in high‐precision radiotherapy techniques such as IMRT and IC/IS BT, the close proximity of tumors to OARs may limit the safe delivery of doses required for tumor control. In cervical cancer brachytherapy, image‐guided hyaluronate gel injection into the rectovaginal and vesicouterine spaces can temporarily separate the target from adjacent OARs. A Japanese propensity score‐matched study found that this approach was associated with higher HR‐CTV D90 without increasing bladder or rectal doses [89].

With the same aim of creating physical separation, a bioabsorbable polyglycolic acid tissue spacer was developed in Japan and introduced into clinical practice in 2019 [90]. Surgically placed between the tumor and adjacent normal tissues (Figure 3), this spacer can reduce radiation exposure to OARs. It maintains sufficient thickness during the treatment period and is subsequently absorbed, with near‐complete disappearance within approximately eight months [90, 91]. Initially developed for particle beam therapy for abdominal and pelvic malignancies, its national health insurance coverage in Japan was expanded to radiotherapy more broadly in 2023. Recent reports have described its use in reirradiation for recurrent cervical cancer, including both EBRT [92] and brachytherapy [93].

**FIGURE 3:**
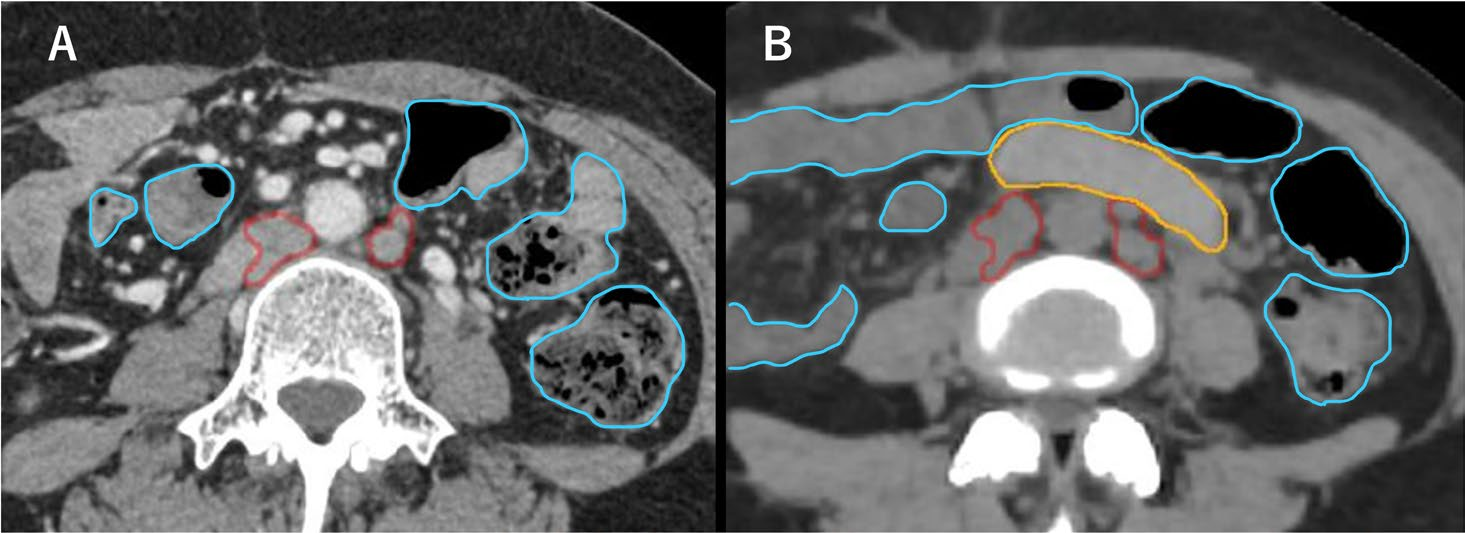
Bioabsorbable tissue spacer placement in a patient with para‐aortic lymph node recurrence after whole‐pelvic concurrent chemoradiotherapy for cervical cancer. The recurrent lymph node, contoured in red, was located at the superior edge of the previous irradiation field. (A) A portion of the bowel, contoured in light blue, was in close proximity to the recurrent lymph node, making it difficult to deliver an adequate dose for definitive radiotherapy without spacer placement. (B) A 15‐mm‐thick bioabsorbable tissue spacer, contoured in yellow, was placed between the recurrent lymph node and the bowel to enable definitive radiotherapy.

Unlike injectable gel spacers, bioabsorbable tissue spacers require surgical placement and carry risks of procedure‐related complications, including infection and ileus. Careful patient selection and close coordination between radiation oncologists and surgeons are therefore essential, as emphasized in the appropriate‐use guidelines issued by the Japanese Society for Radiation Oncology (JASTRO) [94].

Clinical evidence supporting these spacer approaches in cervical cancer remains limited, and further evaluation of its efficacy and safety is warranted. Nevertheless, tissue spacers offer a strategy distinct from increasingly sophisticated imaging and dose optimization: modifying the anatomical relationship between the target and adjacent normal tissues to enable radiotherapy that would otherwise be constrained by anatomy.

### 4.3 Toward Personalization: Multiomics and Radiomics

The Cancer Genome Atlas (TCGA) reported a comprehensive genomic analysis of 228 cervical cancers in 2017 [95]. Since then, genomic studies from diverse populations, including Japan, have further characterized the mutational landscape of cervical cancer and revealed its substantial molecular heterogeneity [96, 97, 98, 99].

However, opportunities to translate these genomic insights into clinical decision‐making remain limited. In a Japanese multicenter study of 724 patients with gynecologic cancers, including 162 with cervical cancer, comprehensive genomic profiling identified actionable alterations in 51.1% of patients, yet only 10.1% received genomically matched therapy [100]. Despite these limitations, molecularly guided treatments, such as HER2‐targeted therapy for gastric‐type adenocarcinoma of the cervix, are emerging as promising therapeutic options [101]. These advances suggest that precision medicine incorporating genomic information into treatment selection is beginning to enter clinical practice.

More recently, multi‐omics approaches incorporating proteomic and other molecular data have gained increasing attention. Proteogenomic characterization may enable the classification of cervical cancer into biologically distinct subtypes and inform therapeutic selection [102].

Radiomics represents another approach to treatment personalization by extracting quantitative information from medical images. Radiomics‐based models have increasingly been investigated for tumor characterization, risk stratification, and prediction of treatment outcomes across different imaging modalities and cancer types [103, 104, 105, 106, 107]. In cervical cancer, a multi‐institutional study by JROSG found that combining pretreatment MRI radiomics with clinical parameters improved the prediction of out‐of‐field recurrence after CCRT [108].

Advances in these approaches to individualized assessment may facilitate the selection of systemic therapy and the personalization of radiotherapy dose, target volumes, and combination strategies according to each patient's tumor characteristics and predicted recurrence risk.

## 5 Conclusions

In recent years, radiotherapy for cervical cancer, encompassing both EBRT and brachytherapy, has evolved substantially with advances in image‐guidance technologies. Looking ahead, the integration of AI‐assisted treatment, innovative approaches to overcoming anatomical constraints, and biological information into clinical practice may further transform the field, enabling radiotherapy to evolve from increasingly safe and precise treatment toward truly personalized care tailored to the characteristics of each patient and tumor.

## Author Contributions

**Hitoshi Maemoto:** conceptualization, investigation, writing – original draft, writing – review and editing, visualization, project administration. **Takafumi Toita:** conceptualization, writing – review and editing, supervision. **Akihiro Nishie:** conceptualization, writing – review and editing, supervision, project administration.

## Conflicts of Interest

The authors declare no conflicts of interest.

## Data Availability

Data sharing not applicable to this article as no datasets were generated or analysed during the current study.
